# TGF-β2 Reduces the Cell-Mediated Immunogenicity of Equine MHC-Mismatched Bone Marrow-Derived Mesenchymal Stem Cells Without Altering Immunomodulatory Properties

**DOI:** 10.3389/fcell.2021.628382

**Published:** 2021-02-04

**Authors:** Alix K. Berglund, Julie M. Long, James B. Robertson, Lauren V. Schnabel

**Affiliations:** ^1^Department of Clinical Sciences, College of Veterinary Medicine, North Carolina State University, Raleigh, NC, United States; ^2^Comparative Medicine Institute, North Carolina State University, Raleigh, NC, United States; ^3^Office of Research, College of Veterinary Medicine, North Carolina State University, Raleigh, NC, United States

**Keywords:** mesenchymal stem cell, TGF-β2, cytotoxicity, immunogenicity, major histocompatibility complex, allogeneic

## Abstract

Allogeneic mesenchymal stem cells (MSCs) are a promising cell therapy for treating numerous diseases, but major histocompatibility complex (MHC)-mismatched MSCs can be rejected by the recipient’s immune system. Pre-treating MSCs with transforming growth factor-β2 (TGF-β2) to downregulate surface expression of MHC molecules may enhance the ability of allogeneic MSCs to evade immune responses. We used lymphocyte proliferation assays and ELISAs to analyze the immunomodulatory potential of TGF-β2-treated equine bone marrow-derived MSCs. T cell activation and cytotoxicity assays were then used to measure the *in vitro* cell-mediated immunogenicity. Similar to untreated MSCs, TGF-β2-treated MSCs inhibited T cell proliferation and did not stimulate MHC-mismatched T cells to proliferate. Additionally, similar quantities of prostaglandin E2 and TGF-β1 were detected in assays with untreated and TGF-β2-treated MSCs supporting that TGF-β2-treated MSCs retain their strong immunomodulatory properties *in vitro*. Compared to untreated MSCs, TGF-β2-treated MSCs induced less T cell activation and had reduced cell-mediated cytotoxicity *in vitro*. These results indicate that treating MSCs with TGF-β2 is a promising strategy to reduce the cell-mediated immunogenicity of MHC-mismatched MSCs and facilitate allogeneic MSC therapy.

## Introduction

Mesenchymal stem cells (MSCs) are currently being investigated in clinical trials for the treatment of musculoskeletal, immune-mediated, and degenerative diseases (Squillaro et al., 2016; Galipeau and Sensébé, 2018). The mechanism by which MSCs exert their therapeutic effect appears to be primarily through the secretion of paracrine factors, which inhibit immune responses, promote angiogenesis, reduce apoptosis, and support the recruitment and differentiation of local progenitor cells (da Silva Meirelles et al., 2009; Caplan and Sorrell, 2015). MSCs are strongly immunomodulatory *in vitro*, which initially led investigators to conclude that MSCs were immune privileged and that allogeneic cells could be used without risk of rejection (Le Blanc et al., 2003). However, subsequent *in vivo* studies have demonstrated that major histocompatibility complex (MHC)-mismatched MSCs are in fact recognized and rejected by the recipient immune system (Eliopoulos and Stagg, 2005; Nauta et al., 2006; Badillo et al., 2007; Zangi et al., 2009; Isakova et al., 2014; Pezzanite et al., 2015). Donor MHC I-specific CD8^+^ T cell and cytotoxic alloantibody responses have been detected following *in vivo* administration of allogeneic MSCs (Zangi et al., 2009; Berglund and Schnabel, 2017). Rejection of donor MSCs may lead to increased risk of adverse events and decreased therapeutic potential and must be prevented to realize the full clinical potential of allogeneic MSC therapy (Berglund et al., 2017b).

While *in vivo* studies support that the immunomodulatory properties of MSCs alone cannot prevent allorecognition and rejection *in vivo*, MSCs are rejected more slowly than non-immunomodulatory cells like fibroblasts (Zangi et al., 2009) so they can be considered immune evasive (Ankrum et al., 2014). Additionally, allogeneic MSC therapy is still attractive over autologous therapy as the age and health status of a donor can greatly affect the quality of the cells (Nie et al., 2010; Fafián-Labora et al., 2015). MHC-matching is a labor-intensive process and is not practical in most clinical settings. Manipulation of MHC surface expression on MSCs is a promising strategy for enhancing the immune evasive qualities of MHC-mismatched MSCs and has been shown to promote persistence of allogeneic MSCs in mouse models (de la Garza-Rodea et al., 2011; Huang et al., 2016). We recently published that treatment of equine bone-marrow derived MSCs with 1 ng/ml transforming growth factor-β2 (TGF-β2) significantly downregulated constitutive MHC I surface expression and inhibited IFN-γ-induced MHC I and MHC II expression without altering MSC phenotype or secretion of endogenous TGF-β1 or TGF-β2 (Berglund et al., 2017a). The degree to which MHC I surface expression must be downregulated to prevent T cell activation and rejection of MHC-mismatched MSCs *in vivo* remains unclear.

Mixed lymphocyte reactions (MLRs) and other lymphocyte proliferation assays have traditionally been used to measure MSC immunogenicity (Le Blanc et al., 2003; Tse et al., 2003), but more recent studies have demonstrated that the ability of MSCs to avoid T cell allorecognition and suppress proliferation *in vitro* does not necessarily correlate with the ability to avoid allorecognition *in vivo* (Nauta et al., 2006; Poncelet et al., 2007; Zangi et al., 2009). However, mixed cell cultures or other modified T cell proliferation assays are still useful for measuring the immunomodulatory capabilities and mechanisms of MSCs. For predicting the *in vivo* cell-mediated immunogenicity of MSCs, *in vitro* cytotoxicity assays are more appropriate (Berglund et al., 2017b). As both the immunomodulatory and immune evasive properties are critical for the therapeutic potential of allogeneic MSCs, the goal of this study was to characterize the immunomodulatory properties and cell-mediated immunogenicity of allogeneic TGF-β2-treated equine MSCs. Horses, like humans, are an outbred species and are one of the best available translational models for assessing MSC therapy efficacy for musculoskeletal diseases (Patterson-Kane and Rich, 2014; Kol et al., 2015). Therefore, understanding the immunogenicity of equine MSCs is important for furthering allogeneic MSC therapy in human medicine.

## Materials and Methods

### Horses and MHC-Haplotyping

A total of eight horses were used in this study. All animals were between the ages of 6 and 18 years of age, free of systemic disease as determined by routine physical examinations and bloodwork, free of medication for 48 h prior to use, and non-pregnant. The MHC haplotype of each horse was determined by microsatellite testing as previously described (Table 1; Tallmadge et al., 2010; Tseng et al., 2010). The Institutional Animal Care and Use Committee of North Carolina State University approved the use of horses in these studies.

**TABLE 1:** MHC haplotypes of horses.

		Intra-MHC microsatellite alleles							
		
		Class I		Class II					
			
Horse	ELA haplotype	UMNJH-38	COR110	ABGe9030	EQMHC1	COR112	COR113	UM011	COR114
A	Unclassified	156	221	206	192	243	270	172	249
	Unclassified	156	221	205	194	256	270	172	249
B	A3a	163	207	211	192	254	260	172	243
	A3a	163	207	211	192	254	260	172	243
C	A9a	156	217	215	190	264	272	168	255
	A9a	156	217	215	190	264	272	168	255
D	A2	156	211	209	192	262	268	174	234
	Unclassified	156	221	211	192	262	268	176	247
E	A2	156	211	209	192	262	268	174	234
	Unclassified	156	221	211	192	262	268	176	247
F	A3b	163	207	211	192	262	268	176	247
	A3b	163	207	211	192	262	268	176	247
G	A2	156	211	209	192	262	268	174	234
	Unclassified	156	211	207	190	237	266	179	241
H	A19	156	211	212	190	262	270	184	245
	A9a	156	217	215	190	264	272	169	255

*Equine leukocyte antigen (ELA) haplotypes were assigned by performing PCR amplification of eight polymorphic microsatellite loci and analyzing the length of each amplified fragment.*

### MSC Isolation and Culture

Bone marrow aspirates were collected aseptically from the sternum of horses by using 11-gage Jamshidi bone marrow biopsy needles under standing sedation with local anesthesia. Up to 40 ml of bone marrow was obtained from two sternebrae from each horse and purified via Ficoll-Paque Plus (GE Healthcare, Chicago, IL, United States) gradient centrifugation as previously described (Radcliffe et al., 2010). Untreated and TGF-β2-treated MSCs were cultured as previously described (Berglund et al., 2017a). Isolated bone marrow cells from each horse were plated onto 100 mm tissue culture plates at a density of 300,000 cells/cm^2^. Half of the plates were cultured in normal culture expansion media and half were cultured in media with 1 ng/ml human recombinant TGF-β2 (BioLegend, San Diego, CA, United States). Culture media consisted of 1 g/dl glucose DMEM, 10% fetal bovine serum (FBS) (Atlanta Biolgicals, Flowery Branch, GA, United States), 2 mM L-glutamine, 100 U/ml penicillin and streptomycin, and 1 ng/ml basic fibroblast growth factor (bFGF) (Corning, Inc., Corning, NY, United States). Media were exchanged every 48 h. Cells were passaged at approximately 80% subconfluency by using Accutase cell dissociation solution (Innovative Cell Technologies, Inc., San Diego, CA, United States) and plated at a density of 10,000 cells/cm^2^ for all subsequent passages. All MSCs used in this study were between passages 2 and 4.

Major histocompatibility complex I surface expression on untreated and TGF-β2-treated MSCs from each horse used in this study was compared using an LSRII flow cytometer (BD Biosciences, San Jose, CA, United States). MSCs were labeled with anti-equine MHC I antibody (clone cz3, Antczak Laboratory) at a 1:10 dilution and an APC-conjugated anti-mouse IgG secondary antibody (BD Biosciences) at 1:100 dilution as previously described (Berglund et al., 2017a). MSCs labeled with secondary antibody alone were used as negative controls. MSCs from each horse showed downregulation of MHC I surface expression when treated with TGF-β2 similar to our previously published results (Supplementary Figure 1).

### T Cell Proliferation Assays

Peripheral blood leukocytes (PBLs) were isolated from venous blood by carbonyl iron treatment and Ficoll-Paque Plus gradient centrifugation. Isolated PBLs were re-suspended in lymphocyte media containing RPMI 1640, 10% FBS, 100 U/ml penicillin and streptomycin, and 0.1 mM molecular grade 2-mercaptoethanol.

Modified one-way MLRs were performed in duplicate using responder PBLs with MHC-matched (autologous) or MHC-mismatched stimulator MSCs as previously described (Tse et al., 2003; Schnabel et al., 2014). All MSC stimulator cells used in this assay were MHC II negative. MHC-matched stimulator PBLs were used as negative controls to determine background T cell proliferation and MHC-mismatched stimulator PBLs as positive controls. Untreated and TGF-β2-treated stimulator MSCs were plated at 5 × 10^4^ cells/well in a 24-well plate in standard or TGF-β2 MSC media 24 h before adding responder PBLs. MSC wells were washed twice with phosphate buffered saline (PBS) to remove any exogenous TGF-β2 prior to the addition of responder PBLs. Stimulator PBLs were irradiated with 9 Gy at 60 cGy/min using a Varian Novalis TX linear accelerator and 1 × 10^6^ cells/well were added immediately before the addition of responder PBLs. Responder PBLs were labeled with 5 μM 5(6)-carboxyfluorescein diacetate N-succinimidyl ester (CFSE) and 2 × 10^6^ cells were added to each well. Cultures were maintained for 5 days in lymphocyte media at 37°C and 5% CO_2_.

After culture, PBLs were aspirated from the wells and stained with primary mouse anti-horse CD3 antibody (clone UC-F6G, 1:20 dilution, Laboratory of Dr. J. Scott, University of California-Davis, Davis, CA, United States) and a secondary goat anti-mouse IgG-APC antibody (1:100 dilution, BD Biosciences, Franklin Lakes, NJ, United States). 4′,6′-diamidino-2-phenylindole (DAPI) was added to each sample at a concentration of 500 ng/ml 15 min prior to analysis. Samples were analyzed via flow cytometry using an LSRII (BD Biosciences). Proliferation was calculated using CFSE attenuation and the division index of live, CD3^+^ cells in FlowJo v10 (FlowJo LLC, Ashland, OR, United States). All results were reported as the log fold change relative to the negative control.

### ELISAs

Supernatant from each well of the MLRs was frozen and stored at −80°C prior to use. ELISAs for human TGF-β1 (Promega, Madison, WI, United States) and prostaglandin E2 (PGE2) (Enzo Life Sciences, Inc., Farmingdale, NY, United States) were performed per manufacturer’s instructions. For PGE2 analysis, supernatant from MLRs were diluted 1:2 for reactions with PBL stimulator cells or 1:100 for reactions with MSC stimulator cells in reagent diluent.

### T Cell Activation Assays

Major histocompatibility complex-specific effector cells were generated in mixed leukocyte reactions as previously described (Noronha and Antczak, 2012). Briefly, 50 × 10^6^ PBLs were irradiated with 9 Gy and co-cultured with 100 × 10^6^ responder PBLs in a T175 flask upright for 7 days. Cultures were maintained in media containing 60% AIM-V, 30% RMPI 1640, 10% FBS, 2 mM L-glutamine, 100 U/ml penicillin and streptomycin, 1X non-essential amino acids, 0.5X sodium pyruvate, 0.1 mM molecular grade 2-mercaptoethanol, and 50 U/ml human recombinant IL-2 (PeproTech, Rocky Hill, NJ, United States) at a concentration of 2.5 × 10^6^ cells/ml. On day 7, cultures were re-stimulated with fresh irradiated stimulator cells that were plated at half the density of the surviving responder cells. All cultures were maintained for a total of 10 days and half of the culture media was exchanged for fresh media every 48 h.

2 × 10^6^ effector cells and 5 × 10^4^ MSC target cells were added to 24-well plates. Co-cultures were incubated for 24 h in effector cell media at 37°C and 5% CO_2_. Effector cells were then labeled with primary anti-CD3 antibody as described under T Cell Proliferation Assays or anti-equine CD8 (clone CVS8, 1:80 dilution, Bio-Rad, Hercules, CA, United States).

### *In vitro* Cell-Mediated Cytotoxicity

Major histocompatibility complex-specific effector cells were generated as described above. Untreated and TGF-β2-treated MSC target cells were labeled with 50 μl of chromium-51 (Cr-51) (PerkinElmer, Boston, MA, United States) for 30 min at 37°C and 5% CO_2_. Labeled targets were plated to give effector/target ratios of 50:1 in 200 μl final volume in 96-well round-bottom plates. Spontaneous release control wells contained only target cells and media. 10% Triton X-100 was added to maximum release control wells. All tests were carried out in duplicate. The plates were incubated at 37°C and 5% CO_2_ for 6 h and then centrifuged at 309 × *g* for 3 min. A total of 110 μl of supernatant was harvested from each well and mixed with Ultima Gold scintillation cocktail (PerkinElmer). Cr-51 activity was measured with a Tri-Carb 2900 TR scintillation counter (PerkinElmer) as counts per minute (cpm) over 2 min. Percent cytotoxicity was calculated as % = (experimental cpm−spontaneous cpm)/(maximum cpm−spontaneous cpm) × 100. The percent cytotoxicity for the duplicate wells was then averaged and reported.

### Statistical Analysis

Data from the T cell proliferation assays were normalized by log transformation and analyzed with analysis of covariance (ANCOVA) with horse as covariate. When ANCOVA indicated significant differences (*p* < 0.05), a Tukey’s test was used for multiple comparisons of individual means. Differences in CD3 and CD8 surface expression and percent cytotoxicity were analyzed using paired *t*-tests on the response to treatments matched by donor horse and MHC-matched or mismatched with a null hypothesis of no difference. All analyses were performed using JMP Pro 14 (SAS Institute Inc., Cary, NC, United States) or R (R Core Team, Vienna, Austria).

## Results

### TGF-β2-Treated MSCs Inhibit MHC-Mismatched T Cell Proliferation *in vitro*

**FIGURE 1:**
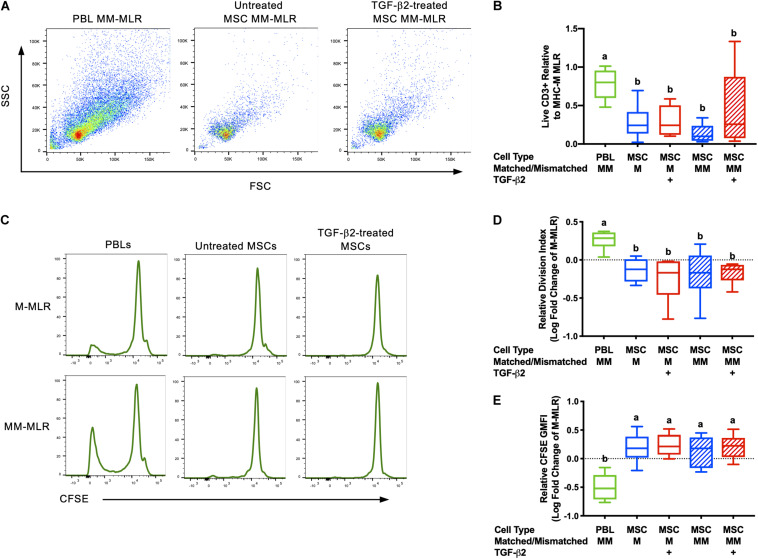
Untreated and TGF-β2-treated MSCs inhibit proliferation of MHC-mismatched T cells *in vitro*. **(A)** Representative dot plots of live, CD3^+^ cells from MLRs with MHC-mismatched PBL, untreated MSC, and TGF-β2-treated MSC stimulator cells. **(B)** Number of live CD3^+^ cells recovered from MLRs relative to the MHC-M MLR with PBL stimulator cells. Data shown are mean ± SD of *n* = 6, *p* = 0.0046 by ANCOVA. Superscript letters indicate significant difference between groups by Tukey’s test. **(C)** Representative histograms depicting CFSE attenuation in responder PBLs cultured with MHC-matched (M) and MHC-mismatched (MM) PBL, untreated, and TGF-β2-treated MSC stimulator cells. **(D)** Proliferation of live, CD3^+^ responder T cells shown as the average log fold change in division index relative to the MHC-M MLR with PBL stimulator cells. Data shown are mean, minimum, and maximum values of *n* = 6, *p* = 0.0025 by ANCOVA. **(E)** Proliferation of live, CD3^+^ responder T cells shown as the average log fold change in CFSE geometric mean fluorescent intensity relative to the MHC-M MLR with PBL stimulator cells. Data shown are mean, minimum, and maximum values for *n* = 6, *p* = 0.0001 by ANCOVA.

Inhibition of allogeneic T cell proliferation *in vitro* is an established characteristic of MSCs, but this ability can be influenced by expression of immunogenic surface molecules and secretion of cytokines (Rasmusson et al., 2005; Carrade Holt et al., 2014; Schnabel et al., 2014). As immunomodulation is critical for both the immune evasive and therapeutic properties of MSCs, the ability of untreated and TGF-β2-treated MSCs to avoid T cell allorecognition and inhibit T cell proliferation was measured using modified one-way MLRs. Significantly fewer live CD3^+^ cells were recovered from assays with MSC stimulator cells compared with the PBL stimulator cells, although there was variation between horses (Figures 1A,B). Background T cell proliferation was detected in the negative control (MHC-matched PBL stimulator cells) by the presence of live CD3^+^CFSE^low^ cells and as expected, an increased number of live CD3^+^ CFSE^low^ cells were detected in the positive control (MHC-mismatched with PBL stimulator cells) (Figure 1C). There were fewer CFSE^low^ CD3^+^ cells in the MHC-matched and MHC-mismatched lymphocyte cultures with MSC stimulator cells even compared to the negative control (Figure 1C). T cell proliferation was significantly reduced in all MLRs with MSC stimulator cells compared to assays with MHC-mismatched PBL stimulator cells as measured by both the relative division index (Figure 1D) and relative GMFI of proliferating cells (Figure 1E). The relative division index and CFSE GMFI in all MLRs with MSC stimulator cells were lower compared to the negative control so neither untreated nor TGF-β2-treated MSCs induced T cell proliferation and both inhibited non-specific background proliferation. Although there was more variation in the number of live CD3^+^ cells recovered from MHC-mismatched MLRs with TGF-β2-treated MSC stimulator cells, the number of CD3^+^ cells in these assays did not correlate with increased proliferation. There was no significant difference between the untreated or TGF-β2-treated MSC treatment groups for either the relative division index or relative CFSE GMFI demonstrating that untreated and TGF-β2-treated MSCs display similar abilities to evade T cell allorecognition and inhibit proliferation *in vitro*.

### Untreated and TGF-β2-Treated MSCs Secrete Similar Amounts of PGE2 and TGF-β1

**FIGURE 2:**
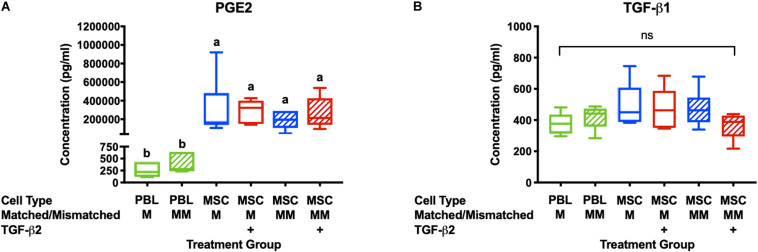
PGE2 and TGF-β1 are secreted in MLRs with PBL and MSC stimulator cells. **(A)** PGE2 concentrations in the supernatant of MLRs as measured by ELISA. Data shown are mean ± SD of *n* = 6, *p* < 0.0001 by ANCOVA. Superscript letters indicate significant difference between groups. **(B)** TGF-β1 concentrations in MLR supernatant as measured by ELISA. Data shown are mean, minimum, and maximum values for *n* = 6, non-significant by ANCOVA.

Prostaglandin E2and TGF-β1 have previously been identified as the major immunomodulatory cytokines secreted by equine MSCs and are also important for the therapeutic properties of MSCs (da Silva Meirelles et al., 2009; Carrade et al., 2012; Carrade Holt et al., 2014). PGE2 concentrations were significantly higher in all cultures with MSC stimulator cells than in wells with PBL stimulator cells and there was no significant difference between PGE2 concentrations in the supernatant from cultures with untreated or TGF-β2-treated MSCs (Figure 2A). There was no significant difference in the concentration of TGF-β1 in the supernatant of any of the MLR treatment groups (Figure 2B). Although we cannot definitively determine if the TGF-β1 is produced by the MSCs or responder PBLs in this assay, these findings are consistent with our previous study that showed TGF-β2-treated MSCs produce similar quantities of TGF-β1 compared to untreated MSCs when cultured alone (14). These results support that TGF-β2 treatment does not affect production or secretion of the main immunomodulatory factors produced by equine bone marrow-derived MSCs.

### TGF-β2-Treated MSCs Induce Less T Cell Activation *in vitro*

**FIGURE 3:**
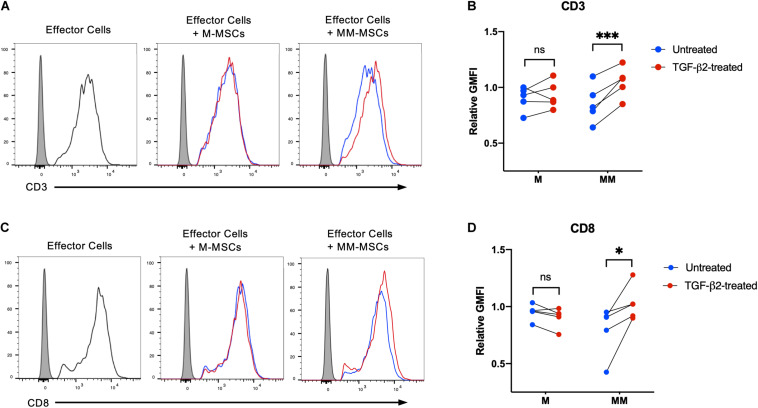
TGF-β2 treatment of MHC-mismatched MSCs prevents activation of T cells. **(A)** Representative histograms of CD3 expression on effector lymphocytes from one horse cultured with MHC-matched or MHC-mismatched untreated or TGF-β2-treated MSCs. **(B)** CD3 expression on effector lymphocytes relative to effector cells incubated without MSCs. MSC donor-paired samples are represented by connected lines for *n* = 5, ***p = 0.0016 by a one-sample *t*-test on each pair. **(C)** Representative histograms of CD8 expression on effector lymphocytes from one horse cultured with MHC-matched or MHC-mismatched untreated or TGF-β2-treated MSCs. **(D)** CD8 expression on effector lymphocytes relative to effector cells incubated without MSCs. MSC donor-paired samples are represented by connected lines for *n* = 5, * *p* = 0.0223 by a one-sample *t*-test on each pair.

Activation of CD8^+^ T cells by MHC I molecules induces downregulation of CD3 and CD8 and secretion of cytolytic effector proteins (DiSanto et al., 1989; Valitutti et al., 1996). The degree of downregulation of CD3 is known to be dependent on the level of T cell receptor engagement (Valitutti et al., 1996). To determine if TGF-β2 treatment prevents MHC-mismatched MSCs from activating effector T cells, untreated and TGF-β2-treated MSCs were co-cultured with effector cells generated in a standard mixed leukocyte reaction prior to analysis of CD3 and CD8 surface expression. Downregulation of both CD3 and CD8 surface expression was detected when effector lymphocytes were cultured with untreated MHC-mismatched MSCs, but not untreated MHC-matched MSCs (Figures 3A,C). Both CD3 and CD8 expression were significantly higher in effector lymphocytes cultured with TGF-β2-treated MSCs compared with untreated MSCs relative to effector cells alone (Figures 3B,D). Changes in CD3 or CD8 surface expression could not be detected with every combination of responders and stimulators demonstrating the natural variation in cell-mediated alloimmune responses. Although not every combination of donors may result in activation of lymphocytes, these results support that T cells were less likely to be activated *in vitro* by TGF-β2-treated MHC-mismatched MSCs compared with untreated MSCs.

### TGF-β2-Treated MSCs Have Reduced T Cell-Mediated Cytotoxicity *in vitro*

**FIGURE 4:**
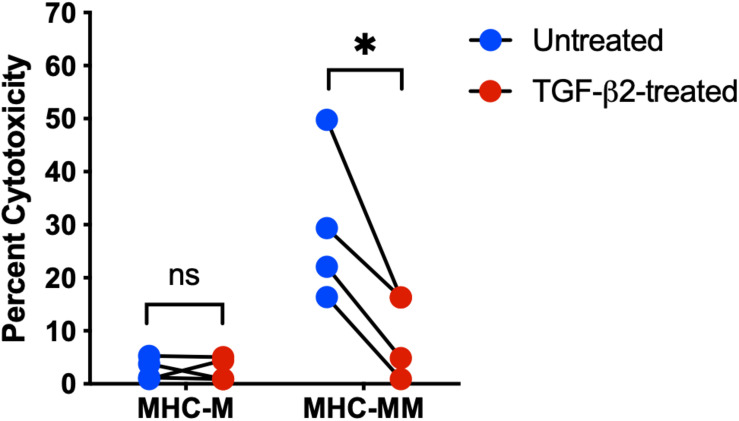
TGF-β2 treatment decreases the cytotoxicity of MHC-mismatched MSCs. Percent cytotoxicity in a standard 6-h chromium-51 release assay for MHC-matched and MHC-mismatched untreated and TGF-β2-treated MSCs. MSC donor-paired samples are represented by connected lines for *n* = 4, *p = 0.0119 by a one-sample *t*-test on each pair.

In a previous study, human MSCs were effective *in vitro* at suppressing activation and proliferation of non-activated T cells, but were unable to inhibit the cytotoxicity of activated cytotoxic T cells (Rasmusson et al., 2003). Untreated and TGF-β2-treated MSC target cells were co-cultured with effector cells and percent cytotoxicity was measured using a Cr-51 release assay. As expected, there was no significant difference in the percent cytotoxicity between untreated and TGF-β2-treated MHC-matched MSCs co-cultured with effector cells (Figure 4). However, percent cytotoxicity was significantly lower for TGF-β2-treated MSCs compared with pair-matched untreated MSCs co-cultured with MHC-mismatched effector cells. As with the T cell activation assays, cytotoxicity was not detected with some combinations of MHC-mismatched effector and target cell donors (Table 2). Although sample size was limited to four MHC-mismatched effector and target cell combinations that demonstrated cytotoxicity, TGF-β2-treatment reduced the cytotoxicity of MSCs from each donor demonstrating that TGF-β2-treated MHC-mismatched MSCs have reduced cell-mediated cytotoxicity compared to untreated MSCs.

**TABLE 2:** MHC haplotypes of responder and stimulator horses for cytotoxicity assays.

Responder ID	Responder haplotype	Stimulator ID	Stimulator haplotype	Cytotoxicity
C	A9a/A9a	E	A2/unclassified	+
E	A2/unclassified	C	A9a/A9a	−
B	A3a/A3a	D	A2/unclassified	−
D	A2/unclassified	B	A3a/A3a	−
A	Unclassified/unclassified	F	A3b/A3b	−
F	A3b/A3b	A	Unclassified/unclassified	−
H	A19/A9a	D	A2/unclassified	+
D	A2/unclassified	H	A19/9a	−
C	A9a/A9a	F	A3b/A3b	+
F	A3b/A3b	C	A9a/A9a	−
H	A19/A9a	G	A2/unclassified	+

*(+) Indicates cytotoxicity was detected for that combination and (−) indicates no cytotoxicity was detected *in vitro*.*

## Discussion

The purpose of this study was to investigate the immunomodulatory properties and cell-mediated immunogenicity of TGF-β2-treated equine bone marrow-derived MSCs. TGF-β2-treated MSCs have significantly reduced MHC I surface expression so we hypothesized that MHC-mismatched MSCs treated with TGF-β2 would retain their immunomodulatory properties and have reduced cell-mediated immunogenicity compared to untreated MHC-mismatched MSCs.

We demonstrated that TGF-β2-treated MHC-mismatched MSCs induce less T cell activation and have significantly reduced cell-mediated cytotoxicity compared to untreated MHC-mismatched MSCs. Additionally, this study supports that untreated MSCs are not innately immune privileged and can activate and be killed by cytotoxic T cells. Previous studies have reported that MSCs inhibit the generation of cytotoxic CD8^+^ T cells, but cannot block the cytotoxic effects of pre-activated effector cells (Rasmusson et al., 2003, 2007). Once injected *in vivo*, MSCs are exposed to the effector and memory CD8^+^ T cells of the recipient’s immune system that do not require co-stimulatory molecules for activation and have a high frequency of cross-reactivity against allogeneic MHC molecules (Whitelegg and Barber, 2004). T cell activation and T cell-mediated killing is dependent on the level of occupancy of the T cell receptor with MHC I surface molecules on the surface of the target (Valitutti et al., 1996). This is consistent with our findings that TGF-β2-treated MHC-mismatched MSCs, which have reduced MHC I surface expression, induce less T cell receptor downregulation and cytotoxicity than untreated MHC-mismatched MSCs *in vitro*. One limitation of this study is that although we determined TGF-β2-treated MSCs had reduced cell-mediated immunogenicity, we cannot definitively state that this was due to decreased MHC I surface expression. It is possible that the TGF-β2 treatment affects other unidentified surface molecules or secretion of cytokines other than PGE2 and TGF-β1 and follow-up studies are in process. An investigation to determine if pre-treatment of MSCs with TGF-β2 is sufficient to delay or prevent rejection of donor MSCs by the recipient immune system *in vivo* is also currently in development.

Major histocompatibility complex -specific effector lymphocytes could not be generated by every combination of MHC-mismatched horses as indicated by the lack of cytotoxicity when effector lymphocytes were co-cultured with untreated MHC-mismatched MSCs. There were also observable differences in the degree of downregulation of CD3 and CD8 and cytotoxicity between responders. We believe both of these observations are most likely due to the natural variation in T cell responses to alloantigens and the MHC haplotypes of the horses used in this study. The affinity and avidity of T cell receptors for MHC molecules is dependent on an individual’s unique T cell repertoire so each responder or recipient may respond more strongly to some MHC haplotypes than others (Obst et al., 2000; Hornell et al., 2003; Stone et al., 2011). Like graft rejections, the degree of an immune response to MSCs is also dependent on the degree of MHC-mismatch between the responder and donor (Qureshi, 1997; Isakova et al., 2014) so *in vivo* studies are needed to compare the immunogenicity of TGF-β2-treated MSCs in a larger and more diverse population. Although cytotoxicity could not be detected from every haplotype combination *in vitro*, this does not support that cytotoxic effector cells against MSCs would not be able to generated *in vivo*. As MSCs are not innately immune privileged and can activate and be killed by lymphocytes, is critical that future pre-clinical and clinical studies using allogeneic MSCs MHC haplotype both donors and recipients and include analysis to detect alloimmune responses.

Modified one-way MLRs or other T cell proliferation assays involving MSCs are noted to be poor predictors of *in vivo* immunogenicity, but are still valuable for measuring the immunomodulatory functions of MSCs. PGE2 and TGF-β1 have both been shown to be important mediators of immune modulation MSCs (English et al., 2009; Carrade Holt et al., 2014). PGE2 suppresses T cell activation and proliferation through inhibition of IL-2 production and transferrin receptor expression (Chouaib et al., 1985) while TGF-β1 also inhibits IL-2 production and is important for the induction of T regulatory cells by MSCs (Brabletz et al., 1993; Hong et al., 2017). The importance of PGE2 to MSC therapeutic efficacy has been demonstrated in models of colitis (Yang et al., 2018), traumatic brain injury (Kota et al., 2017), contact hypersensitivity (Liu et al., 2020), and arthritis (Bouffi et al., 2010). Fewer studies have been conducted investigating the direct therapeutic effects of TGF-β secretion by MSCs, but TGF-β is known to contribute to the proliferation and differential of local stem/progenitor cells, tissue remodeling, extracellular matrix production, and wound healing (Verrecchia and Mauviel, 2002; Xu et al., 2018). Although PGE2 and TGF-β have been identified as particularly important to immunomodulation by MSCs, dozens of other cytokines and paracrine factors contribute to the MSC secretome and may play roles in the immunomodulatory and regenerative properties of MSCs.

It is important to note that while TGF-β2 treatment did not affect the ability of equine MSCs to suppress T cell proliferation in this study, treatment with TGF-β isoforms may negatively affect the immunomodulatory abilities of MSCs in other species. Co-culturing murine MSCs and stimulated lymphocytes in the presence of exogenous TGF-β1 or TGF-β2 reversed the immunosuppressive ability of the murine MSCs due to downregulation of iNOS (27). It is unknown if TGF-β2 affects production of immunomodulatory cytokines in human MSCs, however, targeted strategies other than TGF-β2 treatment could be used to downregulate MHC expression to achieve the same effect in humans without undesirable off target effects. Other strategies that have been published include knocking out MHC I using CRISPR or transfection with viral proteins, although these have their own unintended consequences including susceptibility to NK cell lysis (de la Garza-Rodea et al., 2011; Soland et al., 2012; Shao et al., 2020). Additionally, a dual strategy to reduce MHC I surface expression and increase production of immunomodulatory cytokines via inflammatory cytokine licensing may further enhance the immune evasive properties of MSCs and facilitate allogeneic use.

## Conclusion

In summary, we observed that treating equine bone marrow-derived MSCs with TGF-β2 prior to co-culturing with lymphocytes did not affect the ability of MSCs to suppress T cell proliferation and reduced the cell-mediated immunogenicity of MHC-mismatched MSCs *in vitro*. The results of this study demonstrate a promising approach to reducing the immunogenicity of allogeneic MSCs and improving the safety and efficacy of clinical allogeneic MSC therapy.

## Data Availability Statement

The raw data supporting the conclusions of this article will be made available by the authors, without undue reservation.

## Ethics Statement

The animal study was reviewed and approved by the Institutional Animal Care and Use Committee of North Carolina State University.

## Author Contributions

AB and LS contributed to study conception and design and performed interpretation of the data. AB and JL contributed to the acquisition of the data. AB and JR performed the statistical analysis. AB wrote the manuscript. All authors read and approved the final manuscript.

## Conflict of Interest

The authors declare that the research was conducted in the absence of any commercial or financial relationships that could be construed as a potential conflict of interest.
