# Supplementary figures and images for: TGF-β2 Reduces the Cell-Mediated Immunogenicity of Equine MHC-Mismatched Bone Marrow-Derived Mesenchymal Stem Cells Without Altering Immunomodulatory Properties
Source: Front Cell Dev Biol. 2021 Feb 4;9:628382. doi: 10.3389/fcell.2021.628382 (PMC7889809; doi:10.3389/fcell.2021.628382)

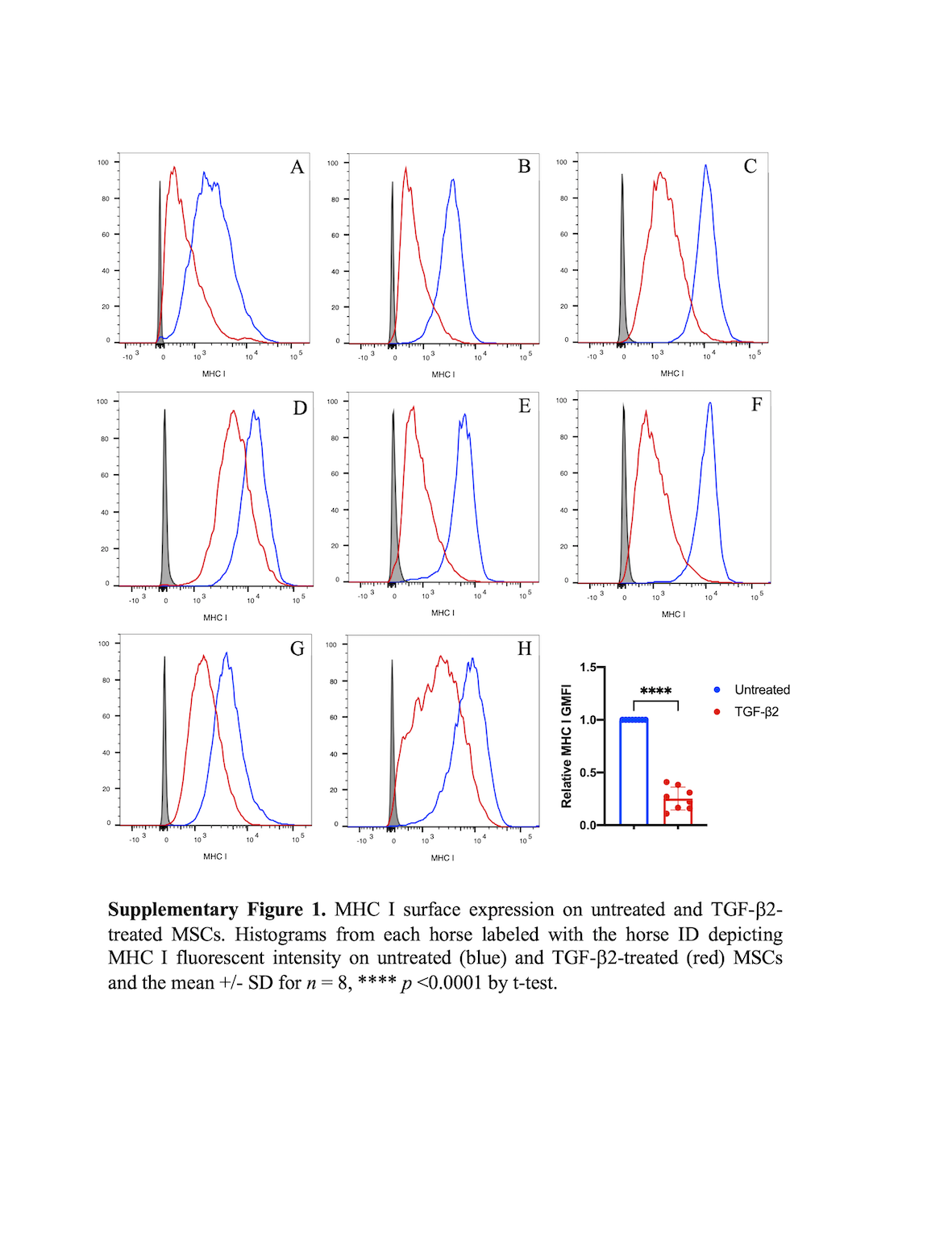

Supplement: Supplementary file 1 [file Image_1.TIFF]
